# An evaluation tool for Age-Friendly and Dementia Friendly Communities

**DOI:** 10.1108/WWOP-11-2017-0032

**Published:** 2018-03-12

**Authors:** Stefanie Buckner, Calum Mattocks, Melanie Rimmer, Louise Lafortune

**Affiliations:** 1 Cambridge Institute of Public Health, University of Cambridge, Cambridge, UK; 2 School of Health and Related Research (ScHARR), University of Sheffield, Sheffield, UK

**Keywords:** Ageing, Age-Friendly Cities, Complex interventions, Dementia Friendly Communities, Evaluation tool, Urbanization

## Abstract

**Purpose:**

The purpose of this paper is to report how an evaluation tool originally developed for Age-Friendly Cities was pilot-tested in the context of the Dementia Friendly Community (DFC) initiative of the city of Sheffield/UK. It presents finding and outputs on which other communities with dementia friendly agendas can draw.

**Design/methodology/approach:**

The original evaluation tool was adapted to a focus on dementia friendliness. Data collection involved scoping conversations, documentary analysis, interviews and group discussions. Following evidence appraisal, Sheffield’s approach to dementia friendliness was assessed. A local steering group was central to the study.

**Findings:**

The evidence indicates areas of strength in Sheffield’s approach to dementia friendliness: involvement of older people; service provision; collaboration; monitoring and evaluation. Scope for improvement was identified around resource allocation, and use of existing guidance on dementia friendliness. Recommendations for policy and practice include enhancing pooling of resources, more detailed recording of resources allocated to dementia-related activity, and collection of evidence on how people affected by dementia have shaped the city’s DFC initiative. Key research outputs are an adaptable logic model and an emerging evaluation framework for DFCs.

**Research limitations/implications:**

The study was a short pilot with limited resources. Its findings and outputs must be considered preliminary.

**Originality/value:**

The findings and outputs provide a basis for further research. The study has suggested key components of an evaluation framework for DFCs. It is informing ongoing work to develop such a framework.

## Introduction

Population ageing and urbanisation are converging global trends. By 2050, the number of people aged 60 years or over is projected to reach almost 2.1 billion worldwide, more than double the 2017 figure of 962 million (United Nations, 2017). Two-thirds of the world’s population are predicted to live in urban areas by then, compared to just under a third in 1950 (United Nations, 2014). In 2006, the World Health Organisation (WHO) responded to these phenomena with its Age-Friendly Cities (AFC) initiative. According to WHO, “[an] age-friendly city encourages active ageing by optimizing opportunities for health, participation and security in order to enhance quality of life as people age” (World Health Organization, 2007, p. 1).

In recent years there has been a rapid increase worldwide in initiatives designed to create Age-Friendly environments. Such initiatives come in different shapes and sizes. While a detailed overview is beyond the scope of this paper, examples are provided by Buckner *et al.* (2017). The proliferation of efforts to enhance the Age-friendliness of different settings has been accompanied by the need for robust evaluation frameworks.

From 2013-2016, researchers in the School for Public Health Research (SPHR) of the National Institute for Health Research (NIHR) in England developed an evidence-based evaluation tool for AFCs that complements existing monitoring and assessment instruments (Buckner *et al.*, 2017). This paper reports on the pilot testing of the tool in Sheffield/UK from November 2016 to March 2017. Researchers worked with a local steering comprising stakeholders from public and voluntary sector agencies and Higher Education, as well as volunteers and older people. The group decided that the research would focus on the city’s Dementia Friendly Community (DFC) initiative as a specific aspect of its Age-Friendly agenda.

While the notion of DFCs is related to the concept of Age-Friendly environments, a debate on the nature of the relationship between both is ongoing (Buckner, 2017; McCann, 2012; Turner and Morken, 2016; Williamson, 2016). As dementia has increasingly come to be recognised as a pressing global health issue (see e.g. Prince *et al.*, 2015), DFCs have emerged as a population level response that emphasises awareness raising and supporting people affected by dementia (those living with the condition and their supporters and carers) in their communities. Similarly to AFCs, DFCs are diverse. They differ in size and ways of working, and they can be geographical communities or communities of interest. Their diversity is reflected in the following broad definition:

Dementia Friendly Communities include, empower and support people affected by dementia and their carers in every aspect of life, from accessing services to using public transport. They can be geographical communities or communities of interest.

They also help empower those whose lives are affected by dementia so that they can remain integrated in society, live as independently as possible and participate actively in decisions that affect their day-to-day lives

(Life Changes Trust, 2015, p. 7).

While globally DFCs are a more recent phenomenon than AFCs, in individual countries such as Japan, efforts to create dementia friendly environments can be traced back to 2004 (Alzheimer’s Disease International, 2016). In the UK, it was the 2012 Prime Minister’s Challenge (Department of Health, 2012) that put DFCs on the agenda. The latter was preceded by research from 2010 to 2012 that informed guidance for local government on how to create DFCs (Local Government Association, 2012). Together with the county of Hampshire, Sheffield was a contributor to this work. The city signed up to a dementia friendly agenda shortly after establishing a Dementia Action Alliance (DAA) in 2013, thus becoming “one of the first cities in the country committed to being a Dementia Friendly Community” (SCCG, n.d.).

The Sheffield steering group decided that the pilot testing of the evaluation tool would involve a dual focus. Together with an overarching assessment of the city’s DFC initiative, a case study of the South Yorkshire Dementia Action Alliances (SYDAA) Dementia Fire and Home Safety Project was carried out. This paper presents findings and outputs that are relevant to both Sheffield and other communities with dementia friendly agendas.

Ethical approval for the study had been obtained from the University of Cambridge.

## Methods

The original AFC evaluation tool identified ten “evidence input areas” – thematic areas where evidence was required for an assessment of initiatives designed to be Age-Friendly (Buckner *et al.*, 2017). Prior to data collection in Sheffield, these were modified slightly to adjust them to a focus on dementia friendliness (Table I).

The tool offers flexibility regarding data sources. In order to ensure appropriateness to the specific context and priorities of a DFC, a stakeholder steering group has a critical role to play in identifying data requirements. Decisions on what evidence will inform an evaluation will furthermore be influenced by the availability of existing data, and by resources for additional data collection.

Documentary evidence supplied by the Sheffield steering group members constituted the main data source across the ten input areas. In addition to scoping conversations with key informants, two semi-structured interviews with stakeholders in dementia-related roles were carried out. The discussions in the steering group meetings (*n*=3) were a further source of information.

**Figure 1:**
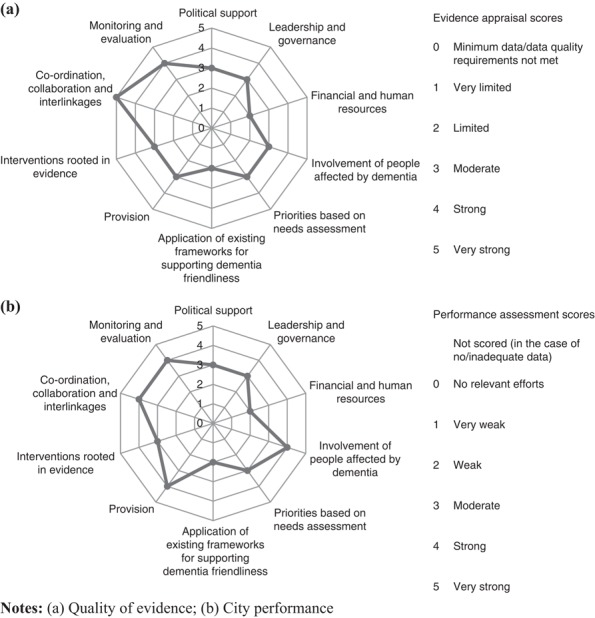
Sheffield’s approach to dementia friendliness

**Figure 2:**
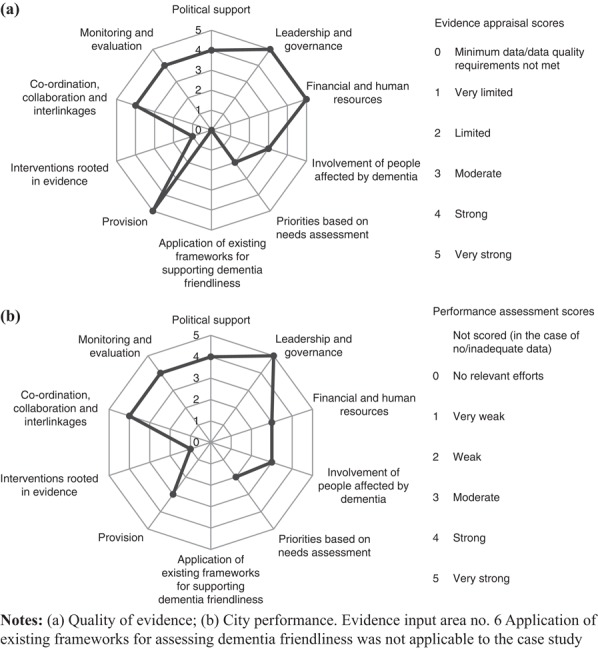
SYDAA Dementia Fire and Home Safety Project

The tool was applied separately to Sheffield’s overall DFC initiative and the case study in a process that is described in detail elsewhere (Buckner *et al.*, 2017). In both instances, evidence from the above sources was recorded for the ten input areas. Appraisal of the quality of the available data preceded assessment of the city’s performance in each input area. The findings from evidence appraisal and performance assessment were captured in a summary score from 0 to 5 (poor/none – very strong) for each input area in both of these components. The resulting ten scores were presented in radar charts for evidence appraisal and performance assessment (Figure 1(a)-(b) and Figure 2(a)-(b)).

Data analysis was carried out by the researchers. The emerging findings were presented to the steering group on an ongoing basis in meetings and a draft report. They were revised in accordance with feedback from the group. It was this iterative process that resulted in an assessment of Sheffield’s dementia friendliness and the subsequent recommendations for policy and practice.

## Findings and outputs

### Sheffield’s approach to dementia friendliness

This section reports the results of the assessment of Sheffield’s work on dementia by evidence input area.

A desire by local politicians to make Sheffield more dementia friendly was identified. However, the means to do so were not always available. While political support thus did not necessarily translate into operational support and implementation, the city’s approach to dementia friendliness was characterised by a clear leadership and governance structure. Key players were the Sheffield Dementia Action Alliance (SDAA) with its cross-agency membership, the Public Health Directorate within Sheffield City Council (SCC), and the Sheffield Clinical Commissioning Group (SCCG). At the same time, the findings suggested that there was scope for responsibilities to be shared more widely, with the implication that the DFC initiative would be owned and promoted by a wider range of partners. A potentially greater role for the transport sector and the local authority’s Planning and Development department in particular became apparent.

The findings indicated that dementia-related activity had been affected less severely by the national context of austerity than other areas of service provision. Nevertheless, Sheffield had experienced substantial funding cuts across relevant sectors. An important response to this had been greater pooling of resources and co-ordination of services by decision makers and providers. An example was the city’s ambitious local implementation of the Better Care Fund, a national integrated health and social care commissioning programme (Local Government Association, 2017; SCCG and SCC, 2015). The aim in Sheffield was that “All commissioning will be completed jointly between Sheffield City Council and the Clinical Commissioning group [sic]” (SCCG and SCC, 2015).

People affected by dementia contributed to various groups and committees that were involved in decision making on the provision and format of services. The available data indicated the existence of involvement structures. Prominent among these was the Sheffield Dementia Involvement Group (SHINDIG) for people with dementia and their carers, which was organised by Sheffield Health and Social Care NHS Foundation Trust and Sheffield Alzheimer’s Society, and through which people affected by dementia became involved in feedback, evaluation and planning of dementia-related services. A key issue that was less clear from the available data was the extent to which the contributions of people affected by dementia translated into action and shaped Sheffield as a DFC.

In 2012, a detailed dementia-specific Health Needs Assessment had been carried out in Sheffield. This continued to inform the city’s DFC initiative, and an update was in preparation at the time of the fieldwork. At the same time, the extent to which the personal experiences and views of service users informed the needs assessment remained unclear. Also, despite elements of good practice, the evidence indicated that a more consistent approach to assessing needs and also assets as a basis for setting priorities remained to be adopted across Sheffield. An important observation was made by a stakeholder with regards to Sheffield’s People Keeping Well in their Community programme. Part of the city’s Better Care Fund, the latter was a major community-based health and wellbeing programme that integrated a focus on dementia and involved annual rounds of bidding by service providers (SCCG and SCC, 2015). The Invitations-To-Tender encouraged providers to design services based on communities’ needs, and they supplied demographic information to support bidders. The programme was seen to have triggered a major shift in commissioning away from pre-specified services towards provision tailored to local need. The emphasis on communities’ need was in turn seen to have contributed to the range of dementia-related activities in the city (see below).

Existing assessment frameworks and guidance for DFCs, specifically by the Alzheimer’s Society (Alzheimer’s Society, 2013, 2014-2015; Green and Lakey, 2013), informed dementia-related work in Sheffield. For example, the emphasis in the guidance on involving people with dementia was reflected in the dementia-related components of the People Keeping Well in their Community programme. The Invitations-To-Tender for service provision stipulated co-production. With regards to dementia-related services within the programme, and in line with existing guidance for DFCs, involvement of people with dementia was a key requirement. Yet although instances could be identified where existing frameworks for DFCs informed work in the city, the evidence suggested that certainly at commissioning level, this occurred in a sporadic rather than in a systematic way.

Sheffield offered people affected by dementia a diverse range of services and facilities, such as dementia cafés, tea dances and home fire safety checks. They included activities specifically aimed at people with dementia and/or those supporting and caring for them. In addition, there were many that were part of mainstream provision and which were designated as particularly suitable for people with dementia. Two major gaps remained. The first was services for people newly diagnosed with dementia and their families and carers, and the second was provision targeted at specific groups such as Black and Minority Ethnic (BAME) communities and lesbian, gay, bisexual and transgender (LGBT) persons. Practitioners and decision makers in the city’s health and other sectors demonstrated keen awareness of these gaps. While a desire to address them was apparent, progress seems to have been hampered by resource constraints.

The available data indicated clear appreciation among stakeholders of the importance of rooting dementia-related interventions in the scientific evidence. However, they did not provide any insights into the extent to which this was translated into practice in Sheffield, and evidence of a consistent approach was absent.

The evidence conveyed a clear sense of cross-sector collaboration as a strength in Sheffield’s approach to dementia friendliness. A key structure was the SDAA, which brought together partners from diverse sectors. Numerous joint strategies, initiatives and projects that were specific to dementia or integrated a relevant focus could be identified. A prominent example was a physical activity project for people with early onset dementia for which funding had been secured through a joint effort by the SDAA, Sheffield Alzheimer’s Society and a local facilities management company (Sheffield International Venues), and which had been evaluated by Sheffield Hallam University (Stevenson, n.d.). Collaboration extended beyond Sheffield, as was illustrated by the regional SYDAA Dementia Fire and Home Safety Project. A major challenge that was highlighted by stakeholders concerned the role of specific parts of the city’s health sector in collaboration on dementia. The research revealed a sense that the latter could be more proactive in this area, although constraints generated by resource pressures were appreciated.

The picture of monitoring and evaluation of dementia-related activity was mixed. There was ample evidence of monitoring and evaluation at the level of individual and often small-scale projects, although not all of this work met criteria for scientific rigour. Routine data were being collected (e.g. dementia diagnosis rates). In addition, a variety of indicators of success were being used, including number of Dementia Friends (Alzheimer’s Society, 2017), and involvement of businesses in the DFC initiative. While there was no shortage of monitoring and evaluation activity, there had been no previous evaluation of the city’s overarching DFC programme. Also, the extent to which findings from monitoring and evaluation exercises were acted upon remained unclear.

The above findings are summarised in Figure 1(b). They need to be considered in the context of the quality of the available data, of which a concise overview is presented in Figure 1(a). The scores were arrived at through consultation with the steering group.

### SYDAA Dementia Fire and Home Safety Project – case study

The SYDAA Dementia Fire and Home Safety Project was a two-year initiative by the four South Yorkshire DAAs (Barnsley, Doncaster, Rotherham and Sheffield) and South Yorkshire Fire and Rescue (SYFR) that ended in September 2017. It focussed on promoting fire and home safety for people affected by dementia.

This project was supported strongly by the mayor of one of the four SYDAAs. It had a clear leadership and governance structure, which involved one part-time co-ordinator from each of the four DAAs. While two of them left during the course of the project, an overall regional co-ordinator joined and provided consistent leadership. A steering group was in place that comprised members of each of the four regional DAAs and a representative from Sheffield Community Foundation, a Community Interest Company (CIC) holding the budget. Funding for the project had been committed for two years by the Stronger Safer Communities Reserve, a Fire Authority scheme investing in preventative work in local communities. An additional £2,000 had been provided by South Yorkshire Police for promoting the Herbert Protocol, a tool to help trace persons with dementia who have gone missing (South Yorkshire Police, 2017). Despite these substantial resource commitments, there was a sense that people living with dementia would have benefited from further funding to purchase safety equipment such as cooker hoods with inbuilt sprinklers. Involvement of people affected by dementia in shaping the project had taken various forms. Feedback had been sought at different stages from carers, family and friends including from members of BAME groups. Carers, family and friends had been consulted on the content of animated videos on aspects of dementia and fire and home safety that had been produced as part of the project (SYDAA, 2016, 2017a, b). People affected by dementia had contributed to a dissemination event.

The emphasis of the project on working with carers, families and friends of people with dementia had been specified by the funders. The extent to which this was based on needs assessment remains unclear. The project itself served as a needs assessment when it identified low awareness of the Herbert Protocol amongst South Yorkshire Police and older people. In response, it came to incorporate work with the Police. This resulted in modifications to the questionnaire that forms part of the Protocol, based on feedback from people affected by dementia. In addition, guidance on completing the questionnaire was developed.

Beyond enhanced use of the Herbert Protocol, a number of other activities were part of service provision through the SYDAA Dementia Fire and Home Safety Project. These included referrals to free fire home safety checks that prioritised people living with dementia, distribution of 1,000 smoke alarm testers, awareness sessions on dementia and fire and home safety for public, private and community organisations, and the production of animations on aspects of fire and home safety (SYDAA, 2016, 2017a, b). Despite the substantial amount of work carried out through the project, scope for further provision around fire and home safety in relation to dementia was identified.

The animated videos had been informed by academic research evidence. Beyond this, the data suggested that there had been a gap in research evidence on fire and home safety and dementia during the formative time of the project. They indicated that the work could have been grounded more firmly in scientific evidence at its later stages, as the time when such evidence was increasingly becoming available.

The project was fundamentally collaborative. It was run in partnership between four DAAs – themselves multi-sector collaborative bodies – and the SYFR. Engagement with organisations and carers, friends and family of people with dementia defined its approach.

Evaluation had been integral to the project, and an evaluation subgroup had been set up. SYFR had monitored take-up of home safety checks by households referred to the service, as well as take-up by agencies of training to become referrers to the service. Service user feedback had been collected. The quarterly release of project funding by the CIC had depended on the submission by SYFR of a completed monitoring form detailing project progress, beneficiaries, partnership working and visibility.

The above findings are presented in Figure 2(b), alongside an overview of the quality of the evidence on which they are based (Figure 2(a)). The scores were arrived at through consultation with the steering group.

### Recommendations for policy and practice

The above findings provided a basis for recommendations of concrete ways in which Sheffield might take the work on dementia friendliness forward. The steering group had opportunities to contribute to and review these in meetings and on the basis of a draft report.

First, a stronger evidence base is required than could be collected within the scope of this pilot study. There is a case for an ongoing collaborative effort between the steering group members, other stakeholders (including people affected by dementia) and researchers to collate further data in which a robust assessment of Sheffield’s performance on dementia friendliness can be grounded.

The scores in Figure 1(b) suggest that Sheffield performed well across the various input areas, with strengths in a number of areas. The latter include input area no. 9: Co-ordination, collaboration and interlinkages. There is a case for exploring the potential of even stronger collaboration in areas where scope for additional efforts was identified, such as in input area no. 3: Financial and human resources. For example, pooling resources by different agencies – beyond the extent to which this was already occurring – might strengthen the resource context of the city’s DFC initiative.

With regards to input area no. 3: Financial and human resources, identifying resources that were allocated to dementia-related activity proved difficult. In addition to initiatives that were specifically targeted at people affected by dementia, there were those that were designed for a broader group of users yet which were commonly accessed by people affected by dementia. This welcome development indicates the importance of more detailed recording of resource allocation, accompanied by clear definitions of the kinds of activities whose resourcing needs to be captured in relation to a city’s DFC initiative.

The involvement and contributions of people affected by dementia, captured by input area no. 4, are commonly emphasised in the literature as fundamental to a DFC (The British Standards Institution, 2015; Green and Lakey, 2013; Hare and Dean, 2015). Robust evidence not only of the nature of such contributions, but also of their impact, is critical for an assessment of a DFC. The gap around this in the data from Sheffield points to a need for systematic monitoring to demonstrate how the contributions of people affected by dementia have shaped the city’s DFC initiative.

### A logic model for DFCs

**Figure 3:**
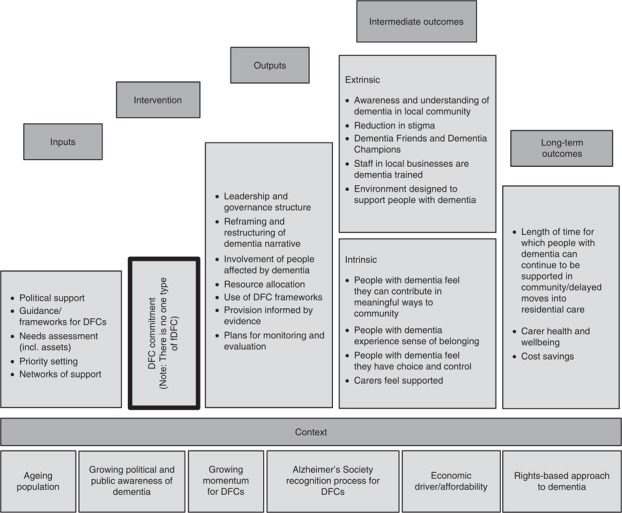
Logic model: Dementia Friendly Communities

A further output from the research in Sheffield was an emerging logic model for DFCs. Logic models are diagrams that provide a simplified overview of a complex system such as an AFC or a DFC. They capture the structures, mechanisms and processes involved (Moore *et al.*, n.d.). Prior to the work in Sheffield, a logic model for AFCs had been developed (Buckner *et al.*, 2017). The pilot testing of the evaluation tool in the city, combined with insights from the literature, initiated a process of adapting the latter to DFCs. The resulting framework (Figure 3) can be applied to different DFC initiatives to guide their design and planning, their implementation and programme management, and their monitoring and evaluation. It provides a flexible instrument that can be adapted to the specific contexts in which it is used.

## Discussion

The research in Sheffield has indicated both strengths and areas for improvement that the city might want to focus on in its efforts to become more dementia friendly. The findings have enabled concrete recommendations for policy and practice. Pilot testing of the evaluation tool has drawn attention to key areas of focus in the context of dementia friendliness. Beyond service provision and involvement of people affected by dementia as commonly emphasised areas, these include structures (e.g. leadership) and processes (e.g. collaboration) that create the conditions for a DFC.

Through piloting in Sheffield, a tool originally designed for AFCs was adapted to a focus on dementia friendliness. It can be employed flexibly in diverse contexts to reflect specific priorities for evaluation as well as considerations such as capacity and data availability. The tool was piloted in a researcher-led process that involved collaboration with a local steering group. This indicated the critical role of a group of stakeholders to ensure both relevance and efficiency of the way in which the tool was applied in a specific context. Ultimately, the tool is intended to be used in a process that is driven by DFC stakeholders, and which can benefit from partnership working with researchers who contribute evaluation expertise as well as a more detached perspective.

Some limitations to the study must be acknowledged. The research in Sheffield was a five month piloting exercise with limited allocated researcher capacity. In addition to the relatively small amount of data collected, this is reflected in the limited extent to which the original AFC tool has been developed into an evaluation framework specifically for DFC initiatives.

In the tool as it was applied in Sheffield, key areas for improvement have been identified. First, scope remains to enhance its ability to capture different kinds of inequalities within DFCs. As a steering group member pointed out, in a large city such as Sheffield, high scores in some of the ten input areas might disguise inequalities in the same areas at smaller geographical scales. While the different input areas can accommodate a focus on inequalities, they do so in a largely implicit way – an observation also made by Buckner *et al.* (2017) with regards to the original AFC evaluation tool. The tool needs to be adjusted in a way that allows it to capture inequalities within a DFC more explicitly, both in terms of (health) outcomes and the structures and processes that shape such outcomes (such as unequal allocation of resources to different areas of work).

Related to this, the piloting has indicated that the tool can capture evidence on specific groups such as BAME and LGBT communities within DFCs. However, it does not do this in a systematic way, and there is a need for a more explicit focus on specific and minority groups.

Finally, the economic aspects of DFCs are not sufficiently captured by the tool as it was applied in Sheffield. Further work is needed to ensure that the tool can generate more detailed insights into the investments into DFCs, and the extent to which this translates into added value – both from a financial and a societal perspective.

## Conclusions

This paper has presented findings and outputs from the piloting of an evaluation tool that was originally developed for AFCs. While these are based on fieldwork in Sheffield as a specific urban setting, their relevance extends beyond this particular context.

The research has yielded insights into the city’s DFC approach that provide learning opportunities for other communities with a dementia friendly agenda. In addition to site-specific findings, it has generated instruments that are designed to be applied widely. It has given rise to a logic model ready to be adapted to different contexts to shape the development, implementation and evaluation of dementia friendly initiatives. Importantly, the research has resulted in an emerging evaluation framework for DFCs. The latter incorporates attention to the involvement of people affected by dementia and to the provision of services as elements that have traditionally been central to DFCs. It integrates these with a focus on interlinking structures and processes that shape DFCs and their ability to enable people affected by dementia to live well.

Due to the study’s limitations, its findings and outputs must be considered preliminary. At the same time, they provide an important basis for further research. While the number of evaluations of DFCs is growing rapidly, a need remains for an evidence-based evaluation framework capable of capturing the complexities of DFCs that can be applied in different contexts. The research reported here has indicated key areas of focus – inequalities; specific groups within DFCS; economic aspects of DFCs – for such a framework. It is informing ongoing work to develop an evaluation tool that will help to arrive at a better understanding of how DFCs can enable people affected by dementia to live well (East of England Collaboration for Leadership in Applied Health Research and Care, 2017).

## Figures and Tables

**Table I:** An emerging evaluation tool for DFCs

Evidence input areas	Definitions
1. Political support	Backing (verbal and/or practical) from key political players locally – e.g. mayor, councillors, parties
2. Leadership and governance	Structures and roles for strategic overview and management
3. Financial and human resources	Commitment of funding, material means, staff, volunteers, investment in staff and volunteers
4. Involvement of people affected by dementia	Instrumental roles and contributions from people affected by dementia (those living with the condition and their supporters and carers). Includes available structures, nature of structures, nature of contributions, impact of contributions
5. Priorities based on needs assessment	Initiatives have been prioritised on the basis of a JSNA and/or other ways of assessing needs
6. Application of existing frameworks for assessing dementia friendliness	Use by the city of existing guidance, e.g. by Alzheimer’s Society, to inform its work on dementia friendliness
7. Provision	Availability of relevant services and facilities, including consistency (e.g. geographical coverage) and continuity (availability and personnel), and consideration of issues around uptake
8. Interventions rooted in evidence	Scientific evidence has been consulted and interventions have been based on the available evidence
9. Co-ordination, collaboration and interlinkages	Partnership working across sectors, co-ordination of relevant activities, and interlinkages between different areas of focus
10. Monitoring and evaluation	Monitoring and evaluation of ongoing and completed work, including plans for monitoring and evaluation and allocation of resources. Nature of monitoring and evaluation. Translation of findings into policy and practice
